# Healthcare utilisation, expenditure, and admission-based mortality associated with paediatric hepatobiliary diseases in Thailand: a national database analysis (2015–2023)

**DOI:** 10.1016/j.lansea.2026.100814

**Published:** 2026-07-08

**Authors:** Songpon Getsuwan, Busara Charoenwat, Chatmanee Lertudomphonwanit, Kaewjai Thepsuthammarat, Suporn Treepongkaruna, Sumitr Sutra

**Affiliations:** a Division of Gastroenterology, Department of Paediatrics, Faculty of Medicine Ramathibodi, Hospital Mahidol University, Bangkok, Thailand; b Ramathibodi Excellence Centre in Organ Transplantation, Faculty of Medicine Ramathibodi Hospital, Mahidol University, Bangkok, Thailand; c Department of Paediatrics, Faculty of Medicine, Khon Kaen University, Khon Kaen, Thailand; d Clinical Epidemiology Unit, Faculty of Medicine, Khon Kaen University, Khon Kaen, Thailand

**Keywords:** Paediatric hepatobiliary diseases, Healthcare utilisation, Hospitalisation, Inpatient mortality, Thailand

## Abstract

**Background:**

Population-level epidemiological data on paediatric hepatobiliary diseases (HBD) are limited in Southeast Asia. We aimed to quantify national trends in healthcare utilisation, expenditure, and admission-based mortality associated with paediatric HBD in Thailand.

**Methods:**

We conducted a retrospective cohort study of hospital admissions among individuals younger than 18 years of age using Thailand's National Health Security Office (NHSO) database from 2015 to 2023. HBD were identified using ICD-10-TM codes. To demonstrate the burden of HBD, we analysed admission rates (AR), length of stay (LOS), NHSO payments, and inpatient mortality rate (IMR). Sensitivity analyses excluding the COVID-19 pandemic years were performed.

**Findings:**

Among 60,916 admissions, 31,158 (51·1%) were male, and the median (IQR) age was 10 (3–14) years. The median (IQR) admission rate (AR) was 59·0 (56·1–65·9) per 100,000 population, with an AAPC of −0·61 (95% CI −2·90 to 1·68). Declining trends in AR were observed for several conditions, including acute hepatitis A, acute hepatitis B, hepatic failure, and cirrhosis. Infants had longer LOS, higher healthcare payments, and higher annual mortality (AM) rate than older children (all *p* < 0·0001). Median (IQR) annual healthcare expenditure was 11·333 (11·151–11·550) million USD. Overall AM rate was 52·4 (45·5–53·9) deaths per 1000 admissions and declined over time (AAPC −4·94; 95% CI −7·35 to −2·52), with similar findings in sensitivity analyses. However, mortality related to hepatic failure remained high without significant temporal improvement.

**Interpretation:**

Paediatric HBD continue to impose a substantial burden on Thailand's healthcare system. Although overall inpatient mortality declined significantly over time, persistently high mortality in hepatic failure highlights ongoing gaps in specialised care. Strengthening referral pathways, critical care capacity, and access to liver care services may further improve outcomes for affected children.

**Funding:**

No funding.

**Research in context**

**Evidence before this study**

We searched PubMed, Embase, Scopus, and regional databases (including Thai Index Medicus) for studies published from Jan 1, 2000, to Dec 31, 2025, reporting population-level burden of paediatric hepatobiliary diseases. Search terms combined “paediatric”, “paediatric” OR “children” with “liver disease”, “hepatobiliary disease”, “acute liver failure”, “cirrhosis”, and “biliary atresia”, together with “incidence”, “mortality”, “hospitalisation”, “burden”, or “health expenditure”, and “Southeast Asia” or individual country names. No language restrictions were applied. Most available evidence derives from high-income countries and is largely limited to single-centre cohorts, disease-specific registries, or transplant-based series. National-level analyses from low- and middle-income countries are scarce, and data from Southeast Asia are fragmented. Few studies have quantified nationwide inpatient mortality, service utilisation, or health-system costs attributable to paediatric liver disease. However, the true health system burden due to paediatric hepatobiliary diseases in countries like Thailand remains poorly characterised.

**Added value of this study**

Using Thailand's nationwide Universal Coverage Scheme database, covering approximately 75% of the national population, we provide a comprehensive national assessment of paediatric hepatobiliary disease burden in Thailand. Across a nine-year period, we quantified admission trends, inpatient mortality, healthcare utilisation, and national health expenditure. We show that although these conditions are relatively uncommon, they are associated with substantial mortality, particularly among infants and in hepatic failure. Despite the presence of liver transplantation services, advanced care remains geographically concentrated in the capital, while patients are distributed nationwide, indicating potential inequity in access to specialised paediatric hepatology services. Our findings shift the perspective from rare-disease epidemiology to health-system burden and service distribution.

**Implications of all the available evidence**

Taken together with existing literature, our findings suggest that paediatric hepatobiliary diseases represent a disproportionately high-risk and resource-intensive group of conditions within middle-income health systems. Persistently high mortality in hepatic failure and regional disparities in access to specialised services highlight gaps in referral pathways, early detection, and transplant system capacity. Strengthening regional referral networks, improving early identification of severe liver disease, and expanding specialist services should be prioritised. Comparable national analyses across Southeast Asia are needed to inform coordinated regional policy and ensure equitable access to life-saving paediatric liver care.

## Introduction

Hepatobiliary diseases (HBD) in children include congenital disorders like biliary atresia, choledochal cysts, and cystic liver disease,^1^ as well as acquired liver disorders due to infection, drugs/toxins, metabolic dysfunction, and autoimmunity.^2^ Some children may also experience disease in the hepatobiliary tract, such as cholelithiasis, cholangitis, and cholecystitis.^1^^,^^3^ Paediatric HBD affect both patients and their families and place substantial burden on the healthcare system.^4^

Although the burden of HBD is less in the paediatric population, these diseases have adverse impacts on the children's health. While acute liver failure could lead to multi-organ dysfunction and death,^5^ chronic liver diseases have long-term consequences, such as development of protein-energy malnutrition, growth faltering, and portal hypertension in cirrhosis which could ultimately lead to mortality.^6^^,^^7^ Children with HBD also require intense diagnostic or therapeutic intervention, notably gastrointestinal endoscopy and abdominal paracentesis. End-stage liver disease or acute liver failure may lead to requirement of complex surgical interventions like liver transplantation (LT).^6^

Existing evidence has shown that global prevalence of hepatitis B infection among children younger than 5 years is 0·7–1·6%,^8^ and approximately 3·2 million children and adolescents around the world are infected with hepatitis C virus.^9^ Some national studies have demonstrated the mortality of certain HBD, such as acute liver failure and biliary atresia.^10^^,^^11^ However, most available evidence is derived from specific liver diseases or single-centre studies; consequently, population-level information on healthcare utilisation, expenditure, and mortality remains limited in low- and middle-income countries, particularly in Southeast Asia.

In such settings, hospital admission data provides a pragmatic measure of disease burden, reflecting both access to care and health-system capacity. Administrative health-care databases allow admission-based epidemiology to be evaluated at a national scale, including temporal trends and system performance where disease registries are not available. Therefore, we aimed to quantify national trends in healthcare utilisation, expenditure, and inpatient mortality associated with paediatric HBD in Thailand under the Universal Coverage Scheme (UCS), Thailand's principal health scheme, which has been implemented since 2002 and currently covers approximately 75% of the country's population.^12^ The UCS is supported by a government reimbursement database maintained by the National Health Security Office (NHSO), in which hospital payments are linked to diagnostic coding and claims submission. This structure captures nationwide inpatient utilisation across public hospitals and therefore represents the functioning of the national health system.

## Methods

This retrospective cohort study included hospitalised children and adolescents younger than 18 years, with permission to retrieve data from the NHSO in Thailand from 2015 to 2023. The information was based on discharge summaries from government hospitals across the country and from some private hospitals under NHSO's contract. The UCS is the predominant public health insurance scheme for Thai children and covers majority of the paediatric population, whereas civil servant and private insurance schemes represent substantially smaller and socioeconomically distinct groups. Therefore, NHSO data provides a close approximation of national paediatric inpatient utilisation within the public health system.

The study identified the admission diagnosis using the International Statistical Classification of Diseases and Related Health Problems, 10th Revision, Thai Modification (ICD-10-TM), and procedures using International Statistical Classification of Diseases, 9th Revision, Clinical Modification (ICD-9-CM) codes. Supplementary Table S1 summarises the ICD-10-TM and ICD-9-CM in this report. HBD was defined using the discharge diagnosis. Accordingly, the analysis reflects disease-driven hospitalisations rather than comorbid conditions. The database is an administrative claims database maintained by Thailand's NHSO for reimbursement purposes and subsequently made available for research use. No additional manual exclusions were applied by the investigators beyond the predefined age criteria and diagnostic definitions.

**Fig. 1:**
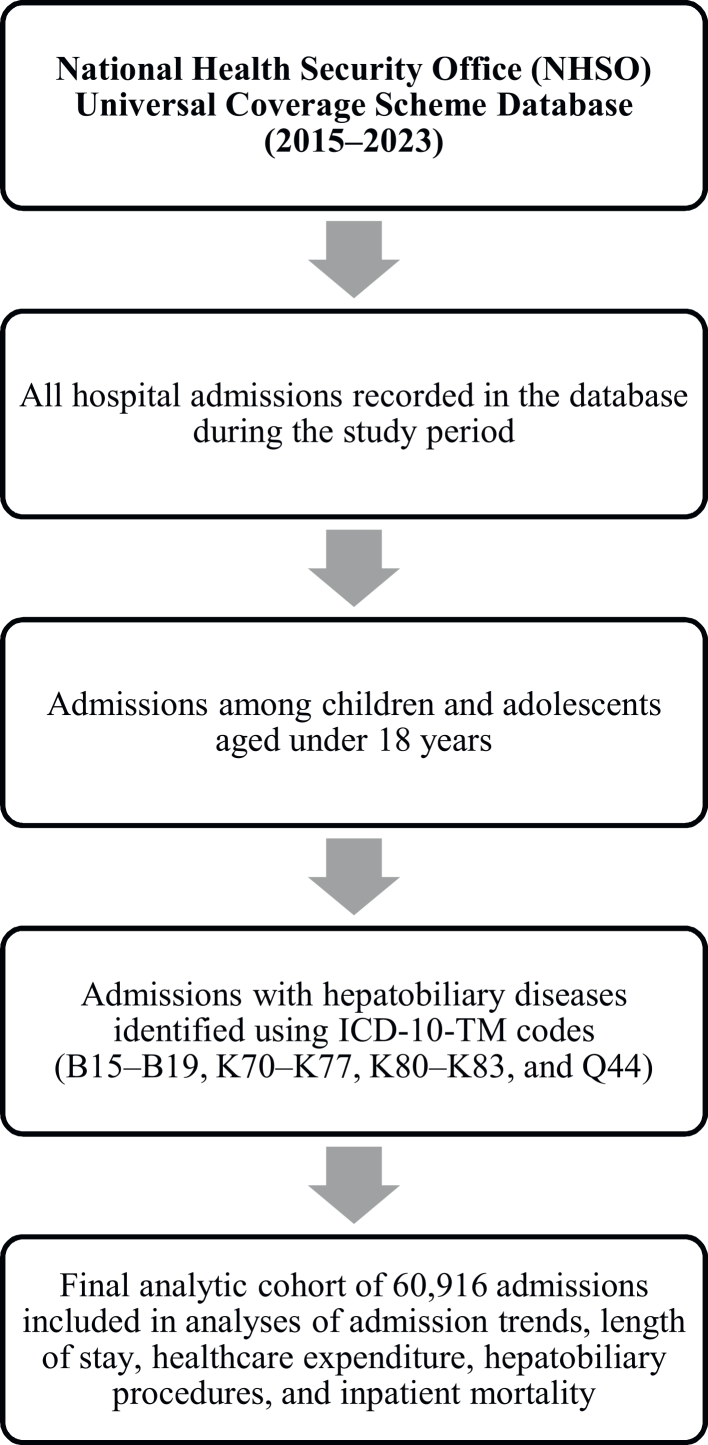
**Flowchart of study population selection.** Hospital admissions among children and adolescents aged younger than 18 years were identified from the National Health Security Office (NHSO) based on Universal Health Coverage Scheme (UCS) database between 2015 and 2023. Admissions with International Statistical Classification of Diseases and Related Health Problems, 10th Revision, Thai Modification (ICD-10-TM) codes corresponding to hepatobiliary diseases were included in the final analytic cohort.

To demonstrate the burdens of HBD, we analysed admission rates, length of stay (LOS), NHSO payments, and inpatient mortality rate (IMR). Demographic data, including age, sex (male or female), hospital region, hospital level, and date of admission, were obtained from the NHSO database. Admission rates were calculated per 100,000 population of all inpatient diagnoses. In-hospital mortality was calculated as admission-based mortality (deaths per 1000 admissions), with each hospitalisation treated as an independent event. Repeated admissions of the same patient were counted separately; thus, estimates reflect in-hospital risk per admission and may underestimate true patient-level case-fatality. The study was reported according to the Strengthening the Reporting of Observational Studies in Epidemiology (STROBE) guidelines (Fig. 1).

### Statistical analysis

The study used the Stata program (StataCorp, Version 17, College Station, Texas, USA) for statistical analysis. The categorical data were presented as frequency (%) and quantitative data were summarised as mean and standard deviation (SD) or median and interquartile range (IQR). Age was categorised into <1 year, 1–5 years, 6–12 years, and 13–17 years. For analyses comparing healthcare utilisation and inpatient mortality, age was additionally dichotomised as <1 year and ≥1 year to distinguish infants from older children. This stratification was pre-specified because infants represent a clinically distinct population with different aetiologies of HBD, healthcare utilisation patterns, and clinical outcomes. The admission number (AN) was described as the total AN per year, and the admission rates were recalculated using population-based denominators and expressed per 100,000 population. LOS was reported as days per admission. IMR was expressed per 1000 admissions. Additionally, the study estimated the average annual percent change (AAPC) to assess temporal trends.

A predefined sensitivity analysis excluding admissions from 2020 to 2021 was conducted to assess the impact of the COVID-19 pandemic; the primary analysis included all study years (2015–2023), and results were compared to evaluate the robustness of temporal trends. Negative binomial or Poisson regression models were applied to assess trends over time, depending on the data distribution. We also performed univariable and multivariable analyses to identify factors associated with IMR, which was additionally expressed per 1000 patient-days to account for time at risk (LOS); the evaluated factors included age group, hospital level, region, and diagnosis.

The NHSO payments were inflation-adjusted to 2023 Thai baht using the Thailand consumer price index and subsequently converted to US dollars using the average 2023 exchange rate reported by the Bank of Thailand (1 USD = 34·52 THB). All costs are presented in 2023 constant prices.^13^ Cost estimates reflect healthcare system payments from the NHSO under UCS, based on diagnosis-related group (DRG) reimbursements per admission, including inpatient services, procedures, and hospital care. Out-of-pocket expenditures and indirect costs were not captured. Statistical inference was based primarily on 95% confidence intervals; two-sided P values < 0·05 were considered supportive evidence of statistical significance.

### Ethics statement

This study received approval from the Ethics Committee for Human Research, Khon Kaen University (Reference number: HE681602; Date: 28 September 2025), and the Human Research Ethics Committee, Faculty of Medicine Ramathibodi Hospital, Mahidol University (Reference number: COA MURA 2025/261; Date: 27 March 2025). The requirement for informed consent was waived because the study involved secondary analysis of de-identified personal data from a nationwide administrative database, with no direct participant contact. It was conducted in accordance with the Helsinki Declaration.

### Role of the funding source

This study received no external funding.

## Results

**Fig. 2:**
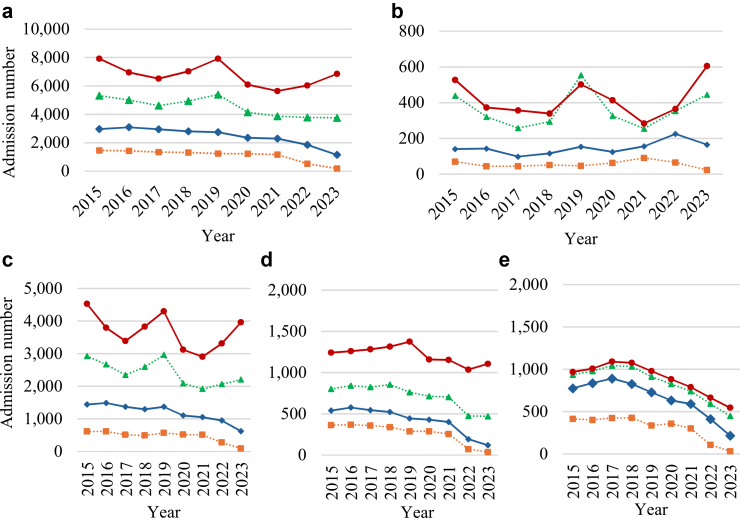
**Distribution of paediatric and adolescent hospitalisations for primary hepatobiliary diseases (a), viral hepatitis (b), diseases of liver (c), disorders of gallbladder and biliary tract (d), congenital malformation of gallbladder, bile ducts, and liver (e) stratified by age group.** Age categories are denoted by shape and colour: orange rectangles indicate children aged <1 year; blue rhombus markers represent 1–5 years; green triangles denote 6–12 years; and red circular dots signify 13–17 years.

Between 2015 and 2023, a total of 60,916 hospital admissions for HBD were recorded among children and adolescents under Thailand's UCS (Table 1). Of these, 31,158 (51·1%) admissions were among males and 29,758 (48·9%) among females; the median age was 10 years (IQR 3–14). Most admissions occurred in adolescents between 13 and 17 years (33·0%), followed by children between 6 and 12 years of age (30·5%) (Fig. 2). Infants accounted for 16·20% of admissions. Geographically, the northeastern region contributed the largest proportion of admissions (36·8%), followed by Bangkok, capital of Thailand (20·8%) (Supplementary Table S2). More than half of hospitalisations were managed in tertiary-level hospitals (54·4%), with a modest shift towards secondary-level care in recent years (Supplementary Figure S1), whereas primary hospitals accounted for 4·7% of cases.

**Table 1:** Baseline characteristics of paediatric hospital admissions for hepatobiliary diseases in Thailand from 2015 to 2023.

Characteristics	Results
**Total number of admissions**	60,916
**Sex, n (%)**	
Male	31,158 (51·1)
Female	29,758 (48·9)
**Age groups, n (%)**	
<1 year	9859 (16·2)
1–5 years	12,329 (20·3)
6–12 years	18,601 (30·5)
13–17 years	20,127 (33·0)
**Geographic regions, n (%)**	
Northeastern region	22,418 (36·8)
Bangkok	12,665 (20·8)
Central region	9190 (15·1)
Southern region	6704 (11·0)
Northern region	4218 (6·9)
Eastern region	3019 (5·0)
Western region	2702 (4·4)
**Hospital levels, n (%)**	
Primary	2836 (4·7)
Secondary	24,253 (39·8)
Tertiary	33,158 (54·4)
Private^a^	669 (1·1)

a Only from hospitals under contract with Thailand's National Health Security Office (NHSO).

From 2015 to 2023, patients younger than 18 years were hospitalised with a median of 6853 admissions per year (6093–7019), corresponding to a population-based admission rate of 59·0 per 100,000 population (56·1–65·9). The overall population-based admission trend did not change significantly over time (AAPC −0·61; 95% CI −2·90 to 1·68). Several specific conditions showed declining temporal trends, including acute hepatitis A, acute hepatitis B, fibrosis and cirrhosis of the liver, other diseases of biliary tract, congenital malformations of gallbladder, bile ducts, and liver, and biliary atresia. Table 2 summarises admissions and temporal trends among paediatric patients with HBD. Most temporal trends observed in the primary analysis remained directionally consistent after excluding pandemic years in sensitivity analysis except for hepatic failure not elsewhere classified (Supplementary Table S3). Chronic viral hepatitis, toxic liver disease and cholelithiasis also demonstrated increasing temporal trends which persisted after excluding 2020–2021.

**Table 2:** Trends of admissions in children and adolescents with diagnosis of hepatobiliary diseases.

Diagnosis (ICD-10)	Number of admissions per year, median (IQR)	Admission rate per 100,000 populations, median (IQR)	AAPC (95% CI)	p value	Length of stay, days per admission, median (IQR)
**Overall hepatobiliary diseases**	6853 (6093–7019)	59·0 (56·1–65·9)	−0·61 (−2·90 to 1·68)	0·60	9·2 (8·6–10·1)
**B15–B19 Viral hepatitis**	929 (800–1178)	8·4 (7·3–9·9)	3·05 (−1·63 to 7·74)	0·20	5·2 (5·0–5·3)
B15 Acute hepatitis A	43 (35–50)	0·40 (0·32–0·42)	−8·29 (−12·89 to −3·68)	0·0004	4·6 (4·1–4·8)
B16 Acute hepatitis B	53 (43–66)	0·5 (0·4–0·6)	−5·83 (−10·11 to −1·56)	0·007	4·8 (4·6–6·6)
B18 Chronic viral hepatitis	125 (122–144)	1·09 (1·06–1·36)	8·05 (5·61 to 10·50)	<0·0001	5·5 (5·3–7·4)
**K70–K77 Diseases of liver**	3797 (3313–3962)	32·3 (29·2–38·0)	−0·59 (−3·66 to 2·49)	0·71	10·0 (8·9–10·7)
K71 Toxic liver disease	309 (268–324)	2·6 (2·4–3·0)	5·20 (1·53 to 8·87)	0·006	15·3 (14·4–16·9)
K72 Hepatic failure	418 (390–455)	3·97 (3·45–4·02)	−3·28 (−6·25 to −0·31)	0·03	13·3 (12·2–14·2)
K74 Fibrosis and cirrhosis	470 (438–493)	4·1 (4·0–4·2)	−3·79 (−6·35 to −1·24)	0·004	10·0 (9·1–10·7)
**K80–K83 Gallbladder and biliary tract disorders**	1243 (1154–1283)	10·7 (10·5–11·0)	−0·56 (−2·02 to 0·91)	0·46	10·8 (9·9–11·7)
K80 Cholelithiasis	470 (432–486)	4·2 (3·9–4·6)	4·94 (2·04 to 7·84)	0·001	5·6 (5·2, 6·0)
K83 Other biliary tract disorders	593 (542–663)	5·3 (5·0–5·7)	−6·18 (−9·26 to −3·10)	0·0001	15·8 (15·7–17·1)
**Q44 Congenital malformations of gallbladder, bile ducts, and liver**	969 (789–1008)	7·3 (6·7–8·1)	−5·39 (−8·17 to −2·61)	0·0001	9·9 (9·6–10·8)
Q44·2 Biliary atresia	716 (547–742)	6·2 (5·3–6·3)	−7·44 (−11·02 to −3·86)	<0·0001	9·4 (9·0–10·3)

AAPC = average annual percentage change. IQR = interquartile range. CI = confidence interval. Admission rates were calculated per 100,000 populations among individuals aged 0–18 years. Diagnoses were classified according to the International Classification of Diseases, 10th Revision (ICD-10). Detailed ICD-10–specific estimates are provided in Supplementary Table S1.

Children and adolescents with HBD reported a median (IQR) LOS of 9·2 (8·6–10·1) days per admission. Infants had substantially longer hospital stays than older children across all diagnostic groups. Overall, median LOS was 18·9 days (IQR 14·0–24·1) in infants compared with 7·0 days (6·3–7·7) in children aged 1 year and older (IRR 2·95, 95% CI 2·40–3·64; p < 0·0001).

Patients with HBD underwent therapeutic endoscopy for oesophageal or gastric varices at a rate of 42·6 per 1000 admissions (IQR 37·3–43·3), with no significant temporal change over time (AAPC −3·17; 95% CI −7·34 to 1·00). Abdominal paracentesis was performed at a median rate of 18·2 per 1000 admissions (IQR 15·9–24·1), with no significant trend in the primary analysis (AAPC −3·43; 95% CI −10·61 to 3·75), but a significant declining trend in the sensitivity analysis. Endoscopic retrograde cholangiopancreatography (ERCP) was performed at a rate of 5·6 per 1000 admissions (IQR 4·5–7·2) and showed a significant increasing trend over time (AAPC 9·33; 95% CI 5·01–13·66); the increase in ERCP was particularly pronounced among patients with diagnosis of congenital malformation of the gallbladder, bile duct, and liver, with a marked rising trend (AAPC 35·38; 95% CI 19·10–51·67). Findings were consistent in sensitivity analyses excluding pandemic years, supporting the robustness of procedural trends (Supplementary Table S5).

The NHSO reimbursed approximately 391·2 million THB, equivalent to 11·3 million USD per year, for paediatric hospitalisations with HBD, with no significant overall change over time (AAPC −0·9; 95% CI −4·01 to 2·09). Declining reimbursement trends were observed for fibrosis and cirrhosis of liver and congenital malformations of the gallbladder, bile ducts, and liver particularly biliary atresia (AAPC −9·05; 95% CI −13·31 to −4·80). In contrast, reimbursement increased for other diseases of liver and viral hepatitis, especially other acute viral hepatitis. Trends are summarised in Table 3 and more details are provided in Supplementary Table S6. Reimbursement per admission was consistently higher among infants than older children across all diagnostic groups. Overall median NHSO payment per admission was USD 2818·3 (IQR 1946·0–4207·4) in infants compared with USD 1217·4 (1052·8–1396·0) in children aged 1 year and older (IRR 3·08, 95% CI 2·37–3·99; p < 0·0001). Similar findings were observed across all major diagnostic groups with similar trend in sensitivity analyses (Supplementary Table S4).

**Table 3:** Trends of payment provided by Thailand's National Health Security Office for hospitalised children and adolescents with diagnosis of hepatobiliary diseases.

Diagnosis (ICD-10)	Payment per year, million USD, median (IQR)	AAPC (95% CI)	p value
Overall hepatobiliary diseases	11·333 (11·151–11·550)	−0·98 (−4·01 to 2·09)	0·53
B15–B19 Viral hepatitis	0·577 (0·468–0·727)	8·36 (2·19–14·54)	0·008
K70–K77 Diseases of liver	7·063 (6·991–7·698)	−0·41 (−3·87 to 3·04)	0·82
K80–K83 Gallbladder and biliary tract disorders	2·106 (1·866–2·165)	−2·76 (−6·44 to 0·92)	0·14
Q44 Congenital malformations of gallbladder, bile ducts, and liver	1·509 (1·392–1·589)	−6·09 (−9·92 to −2·26)	0·002

Values are median (IQR). AAPC = average annual percent change. Payments were inflation-adjusted to 2023 Thai Baht and converted to United States dollars (1 USD = 34·52 THB). Detailed ICD-10–specific estimates are provided in Supplementary Table S1. NHSO = National Health Security Office; USD = United States dollar; THB = Thai Baht.

Overall admission-based mortality among children and adolescents with HBD declined significantly over the study period (AAPC −4·94; 95% CI −7·35 to −2·52), with a similar and slightly greater reduction observed after excluding COVID-19 pandemic years (AAPC −5·82; 95% CI −7·96 to −3·68). The largest reductions were seen in toxic liver disease and fibrosis, cirrhosis of the liver. Detailed mortality trends are summarised in Table 4 and further elaborated in Supplementary Table S7.

**Table 4:** Trends of inpatient mortality among hospitalised children and adolescents with diagnosis of hepatobiliary diseases.

Diagnosis (ICD-10)	Deaths per 1000 admissions, median (IQR)	AAPC (95% CI)	p value
Overall hepatobiliary diseases	52·4 (45·5–53·9)	−4·94 (−7·35 to −2·52)	0·0001
B15–B19 Viral hepatitis	12·9 (11·9–15·3)	2·32 (−4·29 to 8·93)	0·49
K70–K77 Diseases of liver	75·3 (68·7–81·5)	−4·19 (−6·96 to −1·43)	0·003
K80–K83 Gallbladder and biliary tract disorders	20·8 (15·5–23·4)	−7·34 (−12·18 to −2·51)	0·003
Q44 Congenital malformations of gallbladder, bile ducts, and liver	26·6 (23·5–37·2)	−12·36 (−17·27 to −7·46)	<0·0001

Values are median (IQR). Mortality is expressed per 1000 admissions. AAPC = average annual percent change. Detailed ICD-10–specific estimates are provided in Supplementary Table S1.

Several factors were associated with IMR in the univariable analysis, including age, hospital level, region, and diagnostic group. Compared to children between 6 and 12 years, the multivariable analysis demonstrated that patients in other age groups had greater association with inpatient mortality, with the highest incidence rate ratio (IRR) in infants (IRR: 2·19; 95% CI 1·50–3·19). Higher mortality rates were also reported in primary and secondary hospitals when compared to tertiary hospitals (IRR: 3·72; 95% CI 2·05–6·73, and IRR: 1·98; 95% CI 1·27–3·07, respectively). In comparison to admissions in the capital, hospitalisation in the northeastern, southern, and eastern regions was associated with higher mortality rates (IRR: 2·02; 95% CI 1·54–2·65, IRR: 1·86; 95% CI 1·08–3·22, and IRR: 1·64; 95% CI 1·14–2·37, respectively) (Table 5). Among the diagnostic groups, gallbladder and biliary tract disorders, as well as congenital malformations of gallbladder, bile ducts, and liver had lower mortality rates than diseases of liver (IRR: 0·51; 95% CI 0·37–0·71, and IRR: 0·65; 95% CI 0·50–0·84, respectively).

**Table 5:** Univariable and multivariable analysis of inpatient mortality in pediatric hepatobiliary disease.

Factors	Inpatient mortality		Univariable analysis			Multivariable analysis		
	Deaths per 1000 patient-days	(95% CI)	Unadjusted IRR	95% CI	p value	Adjusted IRR	95% CI	p value
**Age**								
6–12 years	15·56	11·66–20·78	Ref.			Ref.		
Less than 1 year	25·40	21·62–29·84	1·97	1·36–2·85	<0·0001	2·19	1·50–3·19	<0·0001
1–5 years	25·05	21·59–29·06	1·90	1·33–2·70	<0·0001	2·05	1·45–2·92	<0·0001
13–17 years	35·59	28·25–44·84	2·50	1·67–3·72	<0·0001	1·89	1·27–2·82	0·002
**Hospital levels**								
Tertiary hospitals	23·12	20·96–25·51	Ref.			Ref.		
Secondary hospitals	62·94	43·16–91·77	5·50	3·02–10·03	<0·0001	1·98	1·27–3·07	0·002
Primary hospitals	132·08	78·22–223·01	2·61	1·69–4·02	<0·0001	3·72	2·05–6·73	<0·0001
**Regions**								
Bangkok metropolitan area	19·49	17·12–22·18	Ref.			Ref.		
Northern region	27·13	20·51–35·90	1·30	0·93–1·81	0·13	1·27	0·91–1·78	0·16
Northeastern region	42·49	34·40–52·49	2·21	1·68–2·91	<0·0001	2·02	1·54–2·65	<0·0001
Central region	14·00	6·67–29·37	0·67	0·30–1·51	0·33	0·62	0·28–1·36	0·24
Southern region	43·39	32·18–58·51	2·20	1·53–3·17	<0·0001	1·64	1·14–2·37	0·008
Eastern region	38·29	23·80–61·59	2·12	1·21–3·72	0·009	1·86	1·08–3·22	0·03
Western region	38·46	20·01–73·92	2·01	0·94–4·27	0·07	1·51	0·74–3·09	0·26
**Primary diagnosis**								
K70–K77 Diseases of liver	31·03	27·49–35·03	Ref.			Ref.		
B15–B19 Viral hepatitis	16·67	2·35–118·32	0·57	0·07–4·70	0·60	0·86	0·11–6·50	0·88
K80–K83 Gallbladder and biliary tract disorders	15·53	11·74–20·55	0·52	0·37–0·73	<0·0001	0·51	0·37–0·71	<0·0001
Q44 Congenital malformations of gallbladder, bile ducts, and liver	20·85	17·51–24·82	0·72	0·56–0·91	0·006	0·65	0·50–0·84	0·001

IRR, incidence rate ratio.

## Discussion

Our study revealed that HBD was not a common diagnosis among hospitalised children. However, the disease burdened the healthcare system in terms of expenditure and contributed to mortality in the paediatric population.

The COVID-19 pandemic may have influenced hospital utilisation patterns and case ascertainment during 2020–2021. However, in sensitivity analyses excluding these years, most temporal trends remained unchanged, although some conditions (e.g., hepatic failure, not elsewhere classified and other acute viral hepatitis) showed differences in statistical significance, suggesting limited but condition-specific impact of the pandemic on observed trends. The observed changes in trends of multiple HBD's among paediatric population in Thailand could be useful to inform future policy and research needs.

We found that overall admission rates did not change significantly, but admission rates for several diagnoses decreased during the study period. The decline in acute hepatitis A remained statistically significant after excluding COVID-19 pandemic years, suggesting that the observed trend was not solely driven by pandemic-related disruptions. Our findings of decreased hepatitis A infection in hospitalised children showed patterns similar to those in a previous study,^14^ suggesting a possible link to improvements in public health and sanitation, which have been associated with lower seroprevalence. According to a systematic review,^15^ Southeast Asian countries also show a trend toward lower endemicity, and Thailand is now considered a country with very low hepatitis A endemicity. Currently, despite hepatitis A vaccine not being included in the country's expanded program on immunisation (EPI), the seroprevalence in children remains relatively low.^16^ A similar pattern was observed for acute hepatitis B, with a persistent decline after sensitivity analysis. Hepatitis B vaccine has been implemented in Thailand's EPI since 1992, and the population born after that has less than 1% of hepatitis B infection prevalence.^17^ Despite an increasing trend of admission rates in the overall chronic viral hepatitis, the change in the rates concerning chronic hepatitis B or C remained insignificant; admission rates for both diagnoses were relatively low compared to other diagnoses in the group in our study. For this reason, further studies are warranted to explore potential explanations for the rising admission rates, particularly those unrelated to hepatitis B or C viruses.

Our study also observed a decreasing trend in admissions for hepatic failure, not elsewhere classified, in the primary analysis; however, this trend was no longer statistically significant after excluding COVID-19 pandemic years, suggesting that it should be interpreted with caution. According to the ICD-10-TM, this diagnosis includes acute and subacute hepatic failure (K72·0) and chronic hepatic failure (K71·2). Dengue infection is the most common cause of paediatric acute liver failure in Thailand.^5^^,^^18^ The incidence of the infection varies between years, with the most common patient age group of adolescents and school-aged children.^19^ Another aetiology includes non-dengue viruses and toxins, such as paracetamol and *Amanita* spp.^5^^,^^18^ However, specific aetiologies of hepatic failure, such as dengue-related liver dysfunction or toxin-induced liver injury (for example, *Amanita phalloides* ingestion), could not be separately identified in this administrative dataset, which limits interpretation of cause-specific trends.

One possible explanation is that the observed decrease in admission rates for hepatic failure in the primary analysis may be partly related to the downtrend in paediatric cirrhosis, thereby lowering the burden of chronic hepatic failure. Biliary atresia, the most common cause of cirrhosis requiring LT in children,^5^ also demonstrated a decreasing trend in admissions that remained significant after sensitivity analysis, indicating a more consistent temporal pattern. As this disease primarily affects infants, the observed decline in admissions for congenital HBD, including biliary atresia, may reflect broader demographic changes, including declining birth rates in Thailand^20^^,^^21^; however, this was not directly examined in our study and awaits further validation.

In contrast to our findings, reports from the United States have demonstrated declining hospitalisation rates for paediatric cholelithiasis.^22^ Cholelithiasis is a common indication for ERCP in children,^23^ and the use of ERCP increased over time in this study, mirroring trends observed internationally.^24^^,^^25^ This increasing procedural use may suggest a possible shift toward centralisation of complex biliary care to tertiary referral centres, where advanced endoscopic procedures are more readily available. Although ERCP is generally considered safe in paediatric population,^26^ younger children—particularly those younger than five years—may have a higher risk of procedure-related complications. In patients with congenital malformations of the gallbladder, bile ducts, and liver, such as choledochal cysts, ERCP should therefore be carefully indicated, particularly when non-invasive investigations are inconclusive or when therapeutic intervention is anticipated.^23^

Moreover, overall reimbursement trends were broadly aligned with admission rates. However, payment for acute viral hepatitis increased despite declining admissions. Because NHSO reimbursement is based on DRG weights and hospital level rather than direct measures of clinical severity, increasing payment per admission could reflect changes in case mix, diagnostic intensity, or referral patterns rather than a true increase in disease burden. The rising cost in acute viral hepatitis might therefore relate to expanded diagnostic investigations, including testing of non-hepatitis viral pathogens. In chronic viral hepatitis, reimbursement remained stable for chronic hepatitis B and hepatitis C. During the study period, national treatment guidelines and indications for antiviral therapy in children remained largely unchanged,^27^^,^^28^ and the UCS did not include paediatric hepatitis C direct-acting antivirals. These policies and treatment structures may partly explain the absence of substantial payment variation over time.

Conversely, reimbursement for other diseases of the liver increased significantly. This category includes heterogeneous sub diagnoses such as metabolic dysfunction associated steatotic liver disease (MASLD), which has shown increasing prevalence globally in children and adolescents,^29^^,^^30^ as well as complications such as portal hypertension requiring complex inpatient management.^8^ Because administrative coding does not permit detailed clinical stratification, we were unable to determine which specific sub-conditions primarily drove the increased reimbursement in this group.

Most importantly, the overall admission-based mortality declined significantly over time in this report. We postulate that health-system strengthening may have contributed to improvements in managing several hepatobiliary conditions, such as earlier referral, improved paediatric intensive care, and expanded financial protection under the UCS, including LT coverage since 2012. However, mortality in hepatic failure did not show a significant reduction and remained markedly high. These patterns were consistent after excluding COVID-19 pandemic years, suggesting that the observed trends were not substantially altered by pandemic-related disruptions. Hepatic failure continues to represent a biologically severe and time-critical subgroup. Acute hepatic failure in Thailand has been reported to arise from systemic infections (including severe dengue), drug-induced liver injury, and immune-mediated disorders, which often present abruptly and progress to multi-organ failure. In such cases, outcomes depend more on rapid stabilisation, critical care capacity, and urgent referral to LT centres than elective planning. Although paediatric LT is available for Thai children, most active centres remain concentrated in the capital city, followed by a single centre in the northern region. Given the narrow therapeutic window of acute liver failure, delays in recognition, referral, or donor availability could have contributed to high short-term mortality despite universal health coverage.

Our multivariable analysis demonstrated significantly higher IMRs of paediatric HBD in the northeastern, southern, and eastern regions compared with Bangkok, as well as in primary and secondary hospitals compared with tertiary centres. These findings may suggest the presence of regional and health-system disparities in outcomes and are consistent with unequal access to specialised hepatology care, paediatric intensive care, and transplant referral pathways among different regions. Several diseases of the liver are complex in the paediatric population, requiring admission to tertiary centres. Unfortunately, not only LT, but hospitals with paediatric gastroenterology and hepatology specialists are clustered in Bangkok, limiting availability in other regions.

In addition to strengthening specialist care and curative services such as LT, preventive strategies are essential to reduce the burden of paediatric HBD. Several conditions identified in this study are potentially preventable, including viral hepatitis through vaccination and public health measures, toxin-related liver injury through improved food safety and health education, and metabolic liver diseases through early detection and lifestyle interventions. Strengthening primary care systems, vaccination coverage, and early screening pathways may help reduce avoidable hospitalisations and downstream healthcare costs.

According to our analysis, infants were particularly vulnerable, with longer hospital stays and higher admission-based mortality, consistent with previous reports.^11^ These findings suggest a need for early detection strategies, strengthened regional referral systems, and equitable access to specialised paediatric liver and critical care services nationwide in young children. The causes of jaundice and liver dysfunction in infants differ substantially from those in older children; however, detailed aetiological classification of neonatal jaundice was not available in this administrative dataset, which primarily captures hepatobiliary diagnoses based on ICD-10-TM coding.

One of the major limitations of this study was its retrospective design. The data was retrieved from discharge summary codes, which may not be accurate in every admission. The number of death cases was also small, undermining the power of statistical analysis. Moreover, our data did not include patients enrolled in other health schemes in Thailand, such as the Social Security Scheme, the Civil Servant Medical Benefit Scheme, or private insurance, which may introduce selection bias and limit the generalisability of the findings to the entire population. Data on race and ethnicity were not collected in the database, precluding analyses by these variables and limiting assessment of population representativeness; these factors may also be influenced by unmeasured socioeconomic and structural determinants. The use of administrative ICD-10-TM coding precluded validation of diagnostic accuracy and limited our ability to identify specific aetiologies of neonatal jaundice and hepatic failure (including dengue-related liver dysfunction and toxin-induced injury [e.g., *Amanita* spp.]), and to report their admission burden and mortality separately. Because the unit of analysis was hospitalisation rather than individual patients, mortality estimates represent admission-based mortality rather than patient-level case-fatality. Repeated admissions of the same patient may therefore lead to overestimation of hospitalisation burden and underestimation of true patient-level mortality. In addition, cost estimates were based on NHSO reimbursement data and reflect healthcare system payments only; out-of-pocket expenditures and indirect costs were not captured, which may lead to underestimation of the true economic burden.

Future work should consider linkage of administrative datasets with clinical registries to enable more detailed disease-specific analyses. Prospective multicentre studies may help to better characterise aetiologies, referral pathways, and access to advanced care. Evaluation of regional variation in access to specialised services, including LT and critical care, may help to better characterise potential disparities in access. In addition, economic evaluations from a broader perspective, incorporating indirect costs, could provide a more complete estimate of disease burden and help plan national strategies. Nevertheless, our results provide insight into paediatric HBD's burdens at a national level for Thailand. Despite low burden, HBD remains an important health problem in the paediatric population contributing towards high hospitalisation rate and mortality. It is important to strengthen hepatology services and referral pathways to improve access to diagnosis and treatment of liver diseases for paediatric population of Thailand.

## Contributors

S.G. and B.C. designed the study. B.C. collected the data. S.G. and B.C. accessed and verified the underlying data and analysed the data. K.T. (statistician) contributed to data interpretation and suggestion on the statistical analyses. S.G. drafted the manuscript and the illustration. C.L., S.T., and S.S. provided critical revisions and conceptual advice. B.C. supervised the study and is the guarantor of the work. All authors read and approved the final manuscript and were responsible for the decision to submit for publication.

## Data sharing statement

The data used in this study were obtained from the National Health Security Office (NHSO) of Thailand. Individual-level data consist of administrative health records and are not publicly available because of legal and privacy restrictions and the absence of permission to redistribute the dataset. The authors did not have access to personally identifiable patient information.

Researchers may request access to the data directly from the NHSO, subject to approval by the data custodians and relevant ethics committees. Data access is governed by the Thai Personal Data Protection Act (PDPA) B.E. 2562 (2019) and NHSO data use regulations.

## Declaration of interests

All authors declare no competing interests.

## Appendix A Supplementary data

Supplementary Tables and Figure

Equitable Partnership Declaration Form

## References

[1] Eiamkulbutr S., Tubjareon C., Sanpavat A., Phewplung T., Srisan N., Sintusek P. (2024). Diseases of bile duct in children. World J Gastroenterol.

[2] Venkatesan P. (2022). New guidance for researching acute hepatitis in children. Lancet Microbe.

[3] Goldman M., Pranikoff T. (2011). Biliary disease in children. Curr Gastroenterol Rep.

[4] Peery A.F., Crockett S.D., Murphy C.C. (2022). Burden and cost of gastrointestinal, liver, and pancreatic diseases in the United States: update 2021. Gastroenterology.

[5] Getsuwan S., Lertudomphonwanit C., Tanpowpong P. (2020). Etiologies, prognostic factors, and outcomes of pediatric acute liver failure in Thailand. Pediatr Gastroenterol Hepatol Nutr.

[6] Forna L., Bozomitu L., Lupu V.V. (2024). Pediatric perspectives on liver cirrhosis: unravelling clinical patterns and therapeutic challenges. J Clin Med.

[7] Milivojevic V., Milosavljevic T. (2020). Burden of gastroduodenal diseases from the global perspective. Curr Treat Options Gastroenterol.

[8] Devarbhavi H., Asrani S.K., Arab J.P., Nartey Y.A., Pose E., Kamath P.S. (2023). Global burden of liver disease: 2023 update. J Hepatol.

[9] Younossi Z.M., Wong G., Anstee Q.M., Henry L. (2023). The global burden of liver disease. Clin Gastroenterol Hepatol.

[10] Kulkarni S., Perez C., Pichardo C. (2015). Use of pediatric health information system database to study the trends in the incidence, management, etiology, and outcomes due to pediatric acute liver failure in the United States from 2008 to 2013. Pediatr Transplant.

[11] Shi Y., Jiang Y.Z., Zhou G.P. (2023). Prognostic factors related to in-hospital death in children with biliary atresia: analysis of a nationwide inpatient database. J Clin Transl Hepatol.

[12] Paek S.C., Meemon N., Wan T.T.H. (2016). Thailand's universal coverage scheme and its impact on health-seeking behavior. Springerplus.

[13] (2023). EC_EI_027: Thailand's Macro economic indicators 1 [Internet]. Bank_of_Thailand. https://app.bot.or.th/BTWS_STAT/statistics/BOTWEBSTAT.aspx?reportID=409&language=ENG.

[14] Sa-nguanmoo P., Posuwan N., Vichaiwattana P. (2016). Declining trend of hepatitis A seroepidemiology in association with improved public health and economic status of Thailand. PLoS One.

[15] Hernandez-Suarez G., Saha D., Lodroño K. (2021). Seroprevalence and incidence of hepatitis A in Southeast Asia: a systematic review. PLoS One.

[16] Kunanitthaworn N., Mueangmo O., Saheng J. (2023). Seroprevalence of hepatitis A virus antibodies among children and adolescents living in Northern Thailand: an implication for hepatitis A immunization. Sci Rep.

[17] Posuwan N., Wasitthankasem R., Pimsing N. (2024). Hepatitis B prevalence in an endemic area of hepatitis C virus: a population-based study implicated in hepatitis elimination in Thailand. J Virus Erad.

[18] Poovorawan Y., Chongsrisawat V., Shafi F. (2013). Acute hepatic failure among hospitalized Thai children. Southeast Asian J Trop Med Public Health.

[19] Thisyakorn U., Saokaew S., Gallagher E. (2022). Epidemiology and costs of dengue in Thailand: a systematic literature review. PLOS Negl Trop Dis.

[20] National_Statistical_Office_Ministry_of_Information_and_Communication_Technology (2015). Statistical year book Thailand 2015. Bangkok: National Statistical Office, Ministry of Information and Communication Technology. https://www.nso.go.th/public/e-book/Statistical-Yearbook/SYB-2015/4/.

[21] National_Statistical_Office_Ministry_of_Digital_Economy_and_Society (2023). Statistical year book Thailand 2023. Bangkok: National Statistical Office, Ministry of Digital Economy and Society. https://www.nso.go.th/public/e-book/Statistical-Yearbook/SYB-2023/.

[22] Agawu A., Kanagawa C., Wong J., Shults J., Feudtner C., Bewtra M. (2023). Pediatric cholelithiasis in the United States: national hospitalization trends, 2006 to 2019. J Pediatr Gastroenterol Nutr.

[23] Tagawa M., Morita A., Imagawa K., Mizokami Y. (2021). Endoscopic retrograde cholangiopancreatography and endoscopic ultrasound in children. Dig Endosc.

[24] Pant C., Sferra T.J., Barth B.A. (2014). Trends in endoscopic retrograde cholangiopancreatography in children within the United States, 2000–2009. J Pediatr Gastroenterol Nutr.

[25] Deptuch K., Szlagatys-Sidorkiewicz A., Koń B., Brzeziński M. (2025). Endoscopic retrograde cholangiopancreatography and post endoscopy cholecystectomies in pediatric population—longitudinal, nationwide data from Poland. J Clin Med.

[26] Hosseini A., Sohouli M.H., Sharifi E. (2023). Indications, success, and adverse event rates of pediatric endoscopic retrograde cholangiopancreatography (ERCP): a systematic review and meta-analysis. BMC Pediatr.

[27] Thai_Association_for_the_Study_of_the_Liver (2015).

[28] Thai_Association_for_the_Study_of_the_Liver (2018).

[29] Hartmann P., Zhang X., Loomba R., Schnabl B. (2023). Global and national prevalence of nonalcoholic fatty liver disease in adolescents: an analysis of the global burden of disease study 2019. Hepatology.

[30] Paik J.M., Kabbara K., Eberly K.E., Younossi Y., Henry L., Younossi Z.M. (2022). Global burden of NAFLD and chronic liver disease among adolescents and young adults. Hepatology.

