# Supplementary material for: Healthcare utilisation, expenditure, and admission-based mortality associated with paediatric hepatobiliary diseases in Thailand: a national database analysis (2015–2023)
Source: Lancet Reg Health Southeast Asia. 2026 Jul 8;52:100814. doi: 10.1016/j.lansea.2026.100814 (PMC13380048; doi:10.1016/j.lansea.2026.100814)
Supplement: Supplementary Tables and Figure [file mmc1.docx]

**Supplementary Appendix**

**Hospitalisation burden and healthcare utilisation of pediatric hepatobiliary diseases in Thailand: a national database analysis (2015–2023)**

## **Supplementary Table**

- Supplementary Table 1. Diagnostic and procedural codes in the study
- Supplementary table 2. Distribution of pediatric and adolescent hospitalizations for hepatobiliary diseases stratified by Thailand’s geographic region
- Supplementary Table 3. Primary and sensitivity analyses of admission trends in children and adolescents with hepatobiliary diseases
- Supplementary table 4. Length of stay and payment by Thailand's National Health Security Office for infants and older children with hepatobiliary diseases
- Supplementary table 5. Detailed ICD-10-specific trends of intervention performed in hospitalized children and adolescents with diagnosis of hepatobiliary diseases
- Supplementary table 6. Detailed trends in annual NHSO payments by ICD-9-CM diagnosis among hospitalized children and adolescents with hepatobiliary diseases
- Supplementary Table 7. Detailed trends in inpatient mortality by ICD-10 diagnosis among hospitalized children and adolescents with hepatobiliary diseases
- Supplementary Figure 1. Distribution of pediatric and adolescent hospitalizations for hepatobiliary diseases stratified by hospital level

## **Supplementary Table 1. Diagnostic and procedural codes in the study**

| **Group** | **Codes** |
| --- | --- |
| **Diseases** | **International Statistical Classification of Diseases and Related Health Problems, 10th Revision, Thai Modification (ICD-10-TM)** |
| Viral hepatitis | - B15, Acute hepatitis A - B16, Acute hepatitis B - B17, Other acute viral hepatitis - B18, Chronic viral hepatitis   - B18.1: Chronic viral hepatitis B with delta-agent   - B18.1: Chronic viral hepatitis B without delta-agent   - B18.2: Chronic viral hepatitis C   - B18.8: Other chronic viral hepatitis - B19, Unspecified viral hepatitis |
| Diseases of liver | - K70, Alcoholic liver disease - K71, Toxic liver disease - K72, Hepatic failure, not elsewhere classified - K73, Chronic hepatitis, not elsewhere classified - K74, Fibrosis and cirrhosis of liver - K75, Other inflammatory liver diseases - K76, Other diseases of liver - K77, Liver disorders in diseases classified elsewhere |
| Disorders of gallbladder and biliary tract | - K80, Cholelithiasis - K81, Cholecystitis - K82, Other diseases of gallbladder - K83, Other diseases of biliary tract - K87, Disorders of gallbladder, biliary tract and pancreas in diseases classified elsewhere |
| Congenital malformations of gallbladder, bile ducts and liver | - Q44.0 Agenesis, aplasia and hypoplasia of gallbladder - Q44.1 Other congenital malformations of gallbladder - Q44.2 Atresia of bile ducts - Q44.3 Congenital stenosis and stricture of bile ducts - Q44.4 Choledochal cyst - Q44.5 Other congenital malformations of bile ducts - Q44.6 Cystic disease of liver - Q44.7 Other congenital malformations of liver |
| **Procedures** | **International Statistical Classification of Diseases, 9th Revision, Clinical Modification (ICD-9-CM)** |
| Therapeutic endoscopy for esophageal or gastric varices | - 42.33, Endoscopic excision or destruction of lesion or tissue of esophagus - 42.91, Ligation of esophageal varices - 44.91, Ligation of gastric varices |
| Endoscopic retrograde cholangiopancreatography | - 51.10, Endoscopic retrograde cholangiopancreatography |
| Abdominal paracentesis | - 54.91, Percutaneous abdominal paracentesis |

## **Supplementary table 2. Distribution of pediatric and adolescent hospitalizations for hepatobiliary diseases stratified by Thailand’s geographic region**

| **Diagnosis / Region** | **2015** | **2017** | **2019** | **2021** | **2023** |
| --- | --- | --- | --- | --- | --- |
| **All-cause inpatient admissions** |  |  |  |  |  |
| Northern | 151,486 | 145,849 | 154,337 | 114,540 | 123,386 |
| Northeastern | 614,504 | 587,684 | 631,645 | 414,352 | 482,422 |
| Central | 340,661 | 321,740 | 331,617 | 239,122 | 241,461 |
| Southern | 327,083 | 333,270 | 352,405 | 286,551 | 264,389 |
| Eastern | 136,673 | 131,171 | 134,321 | 107,856 | 102,434 |
| Western | 97,314 | 91,146 | 94,594 | 75,544 | 72,732 |
| Bangkok metropolitan area | 124,469 | 117,493 | 108,668 | 83,162 | 78,428 |
| **B17: Other acute viral hepatitis** |  |  |  |  |  |
| Northern | 28 | 22 | 42 | 22 | 45 |
| Northeastern | 471 | 240 | 569 | 326 | 534 |
| Central | 97 | 70 | 79 | 63 | 158 |
| Southern | 55 | 48 | 53 | 32 | 53 |
| Eastern | 71 | 25 | 39 | 11 | 52 |
| Western | 64 | 52 | 32 | 45 | 31 |
| Bangkok metropolitan area | 22 | 22 | 20 | 15 | 32 |
| **K72: Hepatic failure, not elsewhere classified** |  |  |  |  |  |
| Northern | 28 | 26 | 29 | 39 | 39 |
| Northeastern | 199 | 103 | 142 | 106 | 125 |
| Central | 142 | 73 | 68 | 58 | 51 |
| Southern | 109 | 93 | 94 | 75 | 70 |
| Eastern | 36 | 24 | 33 | 19 | 35 |
| Western | 39 | 19 | 23 | 28 | 25 |
| Bangkok metropolitan area | 95 | 63 | 61 | 46 | 73 |
| **K75: Other inflammatory liver diseases** |  |  |  |  |  |
| Northern | 133 | 102 | 62 | 94 | 99 |
| Northeastern | 1,015 | 595 | 1,420 | 479 | 1094 |
| Central | 326 | 199 | 278 | 206 | 345 |
| Southern | 270 | 305 | 193 | 88 | 222 |
| Eastern | 141 | 72 | 130 | 67 | 94 |
| Western | 224 | 126 | 90 | 74 | 76 |
| Bangkok metropolitan area | 266 | 144 | 125 | 122 | 174 |
| **Q44.2: Biliary atresia** |  |  |  |  |  |
| Northern | 52 | 60 | 36 | 44 | 19 |
| Northeastern | 130 | 117 | 126 | 116 | 59 |
| Central | 64 | 81 | 74 | 83 | 33 |
| Southern | 54 | 86 | 48 | 75 | 25 |
| Eastern | 9 | 24 | 12 | 24 | 10 |
| Western | 27 | 13 | 4 | 14 | 2 |
| Bangkok metropolitan area | 403 | 393 | 416 | 218 | 167 |

Data are number of admissions. Values are presented as absolute counts with no decimal places. Selected years (2015, 2017, 2019, 2021, and 2023) are shown to represent pre-pandemic, transition, peak disruption, and recovery periods. Only high-burden and clinically relevant hepatobiliary conditions are included. Full annual data are available upon request.

## **Supplementary Table 3. Primary and sensitivity analyses of admission trends in children and adolescents with hepatobiliary diseases**

| **Diagnosis** | **Admission** | | **Primary analysis** | | **Sensitivity analysis*** | |
| --- | --- | --- | --- | --- | --- | --- |
|  | **Number of admissions per year, median (IQR)** | **Admission rate per 100,000 populations, median (IQR)** | **AAPC (95% CI)** | **p value** | **AAPC (95% CI)** | **p value** |
| **Overall hepatobiliary diseases** | 6,853 (6,093–7,019) | 59·0 (56·1–65·9) | −0·61 (−2·90 to 1·68) | 0·60 | 0·14 (−1·97 to 2·25) | 0·90 |
| **B15–B19 Viral hepatitis** | 929 (800–1,178) | 8·4 (7·3–9·9) | 3·05 (−1·63 to 7·74) | 0·20 | 4·14 (−0·81 to 9·10) | 0·10 |
| B15 Acute hepatitis A | 43 (35–50) | 0·40 (0·32–0·42) | −8·29 (−12·89 to −3·68) | 0·0004 | −9·00 (−14·18 to −3·81) | 0·001 |
| B16 Acute hepatitis B | 53 (43–66) | 0·5 (0·4–0·6) | −5·83 (−10·11 to −1·56) | 0·007 | −6·82 (−10·64 to −3·00) | 0·0005 |
| B17 Other acute viral hepatitis | 606 (504–808) | 5·5 (4·3–7·1) | 4·86 (−0·91 to 10·62) | 0·09 | 6·17 (0·06 to 12·29) | 0·048 |
| B18 Chronic viral hepatitis | 125 (122–144) | 1·09 (1·06–1·36) | 8·05 (5·61 to 10·50) | <0·0001 | 7·87 (5·32 to 10·41) | <0·0001 |
| –B18·0 & B18·1 Chronic hepatitis B infection | 9 (5–10) | 0·08 (0·05–0·09) | 1·56 (−7·87 to 10·98) | 0·75 | 4·19 (−4·87 to 13·24) | 0·37 |
| –B18·2 Chronic hepatitis C infection | 3 (2–3) | 0·025 (0·017–0·027) | 2·34 (−13·50 to 18·29) | 0·77 | 3·03 (−14·07 to 20·13) | 0·73 |
| B19 Unspecified viral hepatitis | 96 (61–122) | 0·8 (0·5–1·0) | −5·31 (−15·07 to 4·45) | 0·29 | −2·95 (−12·83 to 6·93) | 0·56 |
| **K70–K77 Diseases of liver** | 3,797 (3,313–3,962) | 32·3 (29·2–38·0) | −0·59 (−3·66 to 2·49) | 0·71 | 0·46 (−2·20 to 3·12) | 0·73 |
| K71 Toxic liver disease | 309 (268–324) | 2·6 (2·4–3·0) | 5·20 (1·53 to 8·87) | 0·006 | 5·84 (2·03 to 9·64) | 0·003 |
| K72 Hepatic failure | 418 (390–455) | 3·97 (3·45–4·02) | −3·28 (−6·25 to −0·31) | 0·03 | −2·60 (−5·56 to 0·35) | 0·08 |
| K73 Chronic hepatitis | 24 (21–25) | 0·22 (0·19–0·23) | −0·57 (−6·52 to 5·37) | 0·85 | 1·40 (−4·14 to 6·94) | 0·62 |
| K74 Fibrosis and cirrhosis | 470 (438–493) | 4·1 (4·0–4·2) | −3·79 (−6·35 to −1·24) | 0·004 | −4·63 (−7·09 to −2·17) | 0·0002 |
| K75 Other inflammatory liver diseases | 1,808 (1,405–2,104) | 15·4 (13·2–19·9) | −1·55 (−6·98 to 3·88) | 0·58 | 0·26 (−4·37 to 4·89) | 0·91 |
| K76 Other diseases of liver | 606 (592–645) | 5·5 (5·0–6·1) | 3·68 (2·18 to 5·17) | <0·0001 | 4·05 (2·55 to 5·54) | <0·0001 |
| **K80–K83 Gallbladder and biliary tract disorders** | 1,243 (1,154–1,283) | 10·7 (10·5–11·0) | −0·56 (−2·02 to 0·91) | 0·46 | −0·48 (−2·22 to 1·27) | 0·59 |
| K80 Cholelithiasis | 470 (432–486) | 4·2 (3·9–4·6) | 4·94 (2·04 to 7·84) | 0·001 | 5·75 (3·02 to 8·48) | <0·0001 |
| K81 Cholecystitis | 147 (130–156) | 1·3 (1·2–1·4) | 1·43 (−1·39 to 4·25) | 0·32 | 2·28 (−0·28 to 4·83) | 0·08 |
| K83 Other biliary tract disorders | 593 (542–663) | 5·3 (5·0–5·7) | −6·18 (−9·26 to −3·10) | 0·0001 | −7·33 (−10·06 to −4·60) | <0·0001 |
| **Q44 Congenital malformations of gallbladder, bile ducts, and liver** | 969 (789–1,008) | 7.3 (6.7-8.1) | −5·39 (−8·17 to −2·61) | 0·0001 | −5·85 (−9·00 to −2·70) | 0·0003 |
| Q44·2 Biliary atresia | 716 (547–742) | 6·2 (5·3–6·3) | −7·44 (−11·02 to −3·86) | <0·0001 | −8·48 (−12·15 to −4·81) | <0·0001 |
| Q44·4 Choledochal cyst | 158 (151–164) | 1·41 (1·39–1·54) | 0·49 (−3·73 to 4·70) | 0·82 | 1·62 (−2·63 to 5·86) | 0·46 |
| Q44·6 Cystic disease of liver | 11 (7–15) | 0·092 (0·061–0·127) | 1·75 (−8·50 to 12·01) | 0·74 | 4·29 (−5·88 to 14·46) | 0·41 |
| Q44·7 Other congenital malformations of liver | 54 (54–66) | 0·49 (0·48–0·61) | −0·17 (−3·75 to 3·42) | 0·93 | −0·85 (−4·75 to 3·06) | 0·67 |

AAPC=average annual percentage change

* Sensitivity analysis excluding years 2020–2021.

## **Supplementary table 4. Length of stay and payment by Thailand's National Health Security Office for infants and older children with hepatobiliary diseases**

| **Diagnosis** | **Length of stay** | | | | **Payment by NHSO per admission (USD)** | | | |
| --- | --- | --- | --- | --- | --- | --- | --- | --- |
|  | **Median (IQR)** | **IRR** | **95%CI** | **p value** | **Median (IQR)** | **IRR** | **95%CI** | **p value** |
| **All hepatobiliary diseases** | |  |  |  |  |  |  |  |
| 1 year and over | 7·0 (6·3–7·7) | Ref |  |  | 1,217·4 (1,052·8–1,396·0) | Ref |  |  |
| Less than 1 year | 18·9 (14·0–24·1) | 2·95 | 2·40 to 3·64 | <0·0001 | 2,818·3 (1,946·0–4,207·4) | 3·08 | 2·37 to 3·99 | <0·0001 |
| ***1) B15-B19, Viral hepatitis*** | |  |  |  |  |  |  |  |
| 1 year and over | 4·9 (4·7–5·1) | Ref |  |  | 552·1 (519·8–645·2) | Ref |  |  |
| Less than 1 year | 9·4 (7·0–10·8) | 1·94 | 1·35 to 2·77 | <0·0001 | 1,097·2 (973·5–1,955·5) | 2·51 | 1·64 to 3·86 | <0·0001 |
| ***2) K70-K77, Diseases of liver*** | |  |  |  |  |  |  |  |
| 1 year and over | 8·2 (6·9–8·4) | Ref |  |  | 1,814·2 (1,640·2–1,823·2) | Ref |  |  |
| Less than 1 year | 23·7 (20·6–27·0) | 3·16 | 2·39 to 4·19 | <0·0001 | 4,176·3 (4,082·6–5,775·1) | 3·20 | 2·41 to 4·24 | <0·0001 |
| ***3) K80-K83, Disorders of gallbladder and biliary tract*** | | | |  |  |  |  |  |
| 1 year and over | 7·1 (6·8–7·5) | Ref |  |  | 1,229·0 (1,171·4–1,288·4) | Ref |  |  |
| Less than 1 year | 22·5 (20·4–25·6) | 3·72 | 2·61 to 5·30 | <0·0001 | 3,380·2 (2,754·6–5,610·6) | 3·75 | 2·52 to 5·58 | <0·0001 |
| ***4) Q44, Congenital malformations of gallbladder, bile ducts and liver*** | | | | | |  |  |  |
| 1 year and over | 7·3 (6·4–7·7) | Ref. |  |  | 1,205.8 (1,161·4-1,341·3) | Ref. |  |  |
| Less than 1 year | 15·4 (15·0–17·7) | 2·67 | 1·87 to 3·82 | <0·0001 | 2,254.1 (2,090·2-3,555·5) | 2·52 | 1·75 to 3·65 | <0·0001 |

Data are median (IQR). P values compare <1 year versus ≥1 year within each diagnostic group. Payments were inflation-adjusted to 2023 THB and converted to USD (1 USD = 34.52 THB). Detailed ICD-10–specific estimates are provided in Supplementary Table 1. Abbreviations: NHSO=National Health Security Office; IQR=interquartile range; THB=Thai Baht; USD=United States dollar.

## **Supplementary table 5. Detailed ICD-9-CM -specific trends of intervention performed in hospitalized children and adolescents with diagnosis of hepatobiliary diseases**

| **Procedures** | **Procedures performed per 1,000 admissions, median (IQR)** | **Primary analysis (All years)** | | **Sensitivity Analysis*** | |
| --- | --- | --- | --- | --- | --- |
|  |  | **AAPC (95%CI)** | **p value** | **AAPC (95% CI)** | **p value** |
| **Therapeutic endoscopy for esophageal or gastric varices** | 42‧6 (37‧3–43‧3) | -3‧17 (-7‧34 to 1‧00) | 0‧14 | -3‧64 (-8‧25 to 0‧96) | 0‧12 |
| - K70-K77, Diseases of liver | 43‧2 (41‧1–46‧3) | -2‧32 (-6‧56 to 1‧91) | 0‧28 | -3‧39 (-7‧56 to 0‧79) | 0‧11 |
| - Q44, Congenital malformations of gallbladder, bile ducts and liver | 106‧0 (90‧0–118‧0) | 0‧48 (-3‧41 to 4‧36) | 0‧81 | 1‧86 (-1‧64 to 5‧35) | 0‧30 |
| **Endoscopic retrograde cholangiopancreatography** | 5‧6 (4‧5–7‧2) | 9‧33 (5‧01 to 13‧66) | <0‧0001 | 9‧73 (5‧35 to 14‧10) | <0‧0001 |
| - K80-K83, Disorders of gallbladder and biliary tract | 26‧9 (21‧0–29‧8) | 5‧88 (-0‧33 to 12‧09) | 0‧06 | 7‧47 (2‧61 to 12‧34) | 0‧003 |
| - Q44, Congenital malformations of gallbladder, bile ducts and liver | 5‧0 (3‧0–7‧0) | 35‧38 (19‧10 to 51‧67) | <0‧0001 | 35‧54 (19‧89 to 51‧20) | <0‧0001 |
| **Abdominal paracentesis** | 18‧2 (15‧9–24‧1) | -3‧43 (-10‧61 to 3‧75) | 0‧35 | -6‧79 (-12‧16 to -1‧41) | 0‧01 |
| - K70-K77, Diseases of liver | 18‧6 (17‧9–24‧0) | -1‧77 (-7‧90 to 4‧37) | 0‧57 | -4‧37 (-9‧17 to 0‧43) | 0‧08 |
| - Q44, Congenital malformations of gallbladder, bile ducts and liver | 37‧6 (33‧7–44‧6) | -3‧00 (-9‧64 to 3‧64) | 0‧38 | -6‧07 (-11‧71 to -0‧44) | 0‧04 |

*Sensitivity analysis excluding 2020 and 2021

Values are median (IQR). AAPC=average annual percent change.

## **Supplementary table 6. Detailed trends in annual NHSO payments by ICD-10 diagnosis among hospitalized children and adolescents with hepatobiliary diseases**

| **Diagnosis** | **Payment per year, million USD, median (IQR)** | **Primary analysis** | | **Sensitivity Analysis*** |  |
| --- | --- | --- | --- | --- | --- |
|  |  | **AAPC (95% CI)** | **p value** | **AAPC (95% CI)** | **p value** |
| **Overall hepatobiliary diseases** | 11‧333 (11‧151–11‧550) | -0‧98 (-4‧01 to 2‧09) | 0‧53 | -0‧06 (-1‧45 to 1‧33) | 0‧93 |
| **B15–B19 Viral hepatitis** | 0‧577 (0‧468–0‧727) | 8‧36 (2‧19 to 14‧54) | 0‧008 | 8‧31 (1‧81 to 14‧80) | 0‧01 |
| B15 Acute hepatitis A | 0‧016 (0‧010–0‧017) | -9‧94 (-42‧60 to 22‧73) | 0‧55 | -8‧46 (-44‧65 to 27‧72) | 0‧65 |
| B16 Acute hepatitis B | 0‧037 (0‧021–0‧038) | 0‧16 (-22‧57 to 22‧90) | 0‧99 | -1‧37 (-27‧42 to 24‧68) | 0‧92 |
| B17 Other acute viral hepatitis | 0‧321 (0‧297–0‧506) | 9‧74 (1‧80 to 17‧67) | 0‧02 | 9‧58 (1‧38 to 17‧79) | 0‧02 |
| B18 Chronic viral hepatitis | 0‧144 (0‧080–0‧205) | 11‧44 (-1‧89 to 24‧77) | 0‧09 | 11‧58 (-2‧54 to 25‧70) | 0‧11 |
| B19 Unspecified viral hepatitis | 0‧034 (0‧027–0‧038) | -0‧88 (-24‧73 to 22‧97) | 0‧94 | -1‧35 (-27‧89 to 25‧19) | 0‧92 |
| **K70–K77 Diseases of liver** | 7‧063 (6‧991–7‧698) | -0‧41 (-3‧87 to 3‧04) | 0‧82 | 0‧78 (-0‧96 to 2‧51) | 0‧38 |
| K71 Toxic liver disease | 0‧839 (0‧778–0‧966) | -1‧90 (-6‧55 to 2‧76) | 0‧43 | -0‧09 (-5‧10 to 4‧92) | 0‧97 |
| K72 Hepatic failure | 2‧081 (1‧931–2‧143) | -1‧89 (-6‧46 to 2‧69) | 0‧42 | -0‧11 (-3‧43 to 3‧20) | 0‧95 |
| K73 Chronic hepatitis | 0‧012 (0‧009–0‧020) | 9‧87 (-32‧11 to 51‧86) | 0‧65 | 10‧94 (-31‧62 to 53‧50) | 0‧61 |
| K74 Fibrosis and cirrhosis | 0‧836 (0‧740–0‧847) | -7‧84 (-12‧55 to -3‧13) | 0‧001 | -8‧45 (-13‧79 to -3‧11) | 0‧002 |
| K75, Other inflammatory liver diseases | 1‧853 (1‧752–1‧926) | -2‧40 (-5‧54 to 0‧75) | 0‧14 | -0‧97 (-4‧34 to 2‧40) | 0‧57 |
| K76 Other diseases of liver | 1‧694 (1‧345–1‧807) | 7‧94 (2‧16 to 13‧72) | 0‧007 | 9‧15 (5‧10 to 13‧20) | <0‧0001 |
| **K80–K83 Gallbladder and biliary tract disorders** | 2‧106 (1‧866–2‧165) | -2‧76 (-6‧44 to 0‧92) | 0‧14 | -1‧73 (-4‧99 to 1‧53) | 0‧30 |
| K80 Cholelithiasis | 0‧465 (0‧384–0‧526) | 1‧78 (-5‧72 to 9‧32) | 0‧33 | 4‧56 (-2‧28 to 11‧40) | 0‧19 |
| K81 Cholecystitis | 0‧120 (0‧088–0‧148) | 9‧61 (-4‧59 to 23‧81) | 0‧19 | 12‧45 (-1‧71 to 26‧61) | 0‧09 |
| K83 Other biliary tract disorders | 1‧476 (1‧336–1‧578) | -5‧51 (-9‧35 to -1‧68) | 0‧005 | -5‧38 (-9‧30 to -1‧47) | 0‧007 |
| **Q44 Congenital malformations of gallbladder, bile ducts, and liver** | 1‧509 (1‧392–1‧589) | -6‧09 (-9‧92 to -2‧26) | 0‧002 | -5‧36 (-9‧19 to -1‧53) | 0‧006 |
| Q44.2 Biliary atresia | 0‧999 (0‧968–1‧039) | -9‧05 (-13‧31 to -4‧80) | <0‧0001 | -9‧50 (-14‧34 to -4‧66) | 0‧0001 |
| Q44.4 Choledochal cyst | 0‧259 (0‧218–0‧325) | -2‧11 (-10‧44 to 6‧23) | 0‧62 | 0‧09 (-8‧82 to 9‧00) | 0‧99 |
| Q44.6 Cystic disease of liver | 0‧044 (0‧026–0‧067) | 13‧54 (-11‧84 to 38‧92) | 0‧30 | 14‧49 (-11‧28 to 40‧25) | 0‧27 |
| Q44.7 Other congenital malformations of liver | 0‧176 (0‧112–0‧184) | -0‧16 (-11‧42 to 11‧09) | 0‧98 | 1‧91 (-9‧91 to 13‧73) | 0‧75 |

* Sensitivity analysis excluding years 2020–2021

Values are median (IQR). AAPC=average annual percent change.

## **Supplementary Table 7. Detailed trends in inpatient mortality by ICD-10 diagnosis among hospitalized children and adolescents with hepatobiliary diseases**

| **Diagnosis (ICD-10)** | **Deaths per 1,000 admissions, median (IQR)** | **Primary analysis (All years)** | | **Sensitivity Analysis*** | |
| --- | --- | --- | --- | --- | --- |
|  |  | **AAPC (95% CI)** | **p value** | **AAPC (95% CI)** | **p value** |
| **Overall hepatobiliary diseases** | 52‧4 (45‧5–53‧9) | -4‧94 (-7‧35 to -2‧52) | 0‧0001 | -5‧82 (-7‧96 to -3‧68) | <0‧0001 |
| **B15-B19, Viral hepatitis** | 12‧9 (11‧9–15‧3) | 2‧32 (-4‧29 to 8‧93) | 0‧49 | 0‧51 (-6‧42 to 7‧44) | 0‧89 |
| **K70-K77, Diseases of liver** | 75‧3 (68‧7–81‧5) | -4‧19 (-6‧96 to -1‧43) | 0‧003 | -5‧28 (-7‧67 to -2‧88) | <0‧0001 |
| K71, Toxic liver disease | 32‧4 (21‧6–46‧0) | -13‧73 (-21‧93 to -5‧52) | 0‧001 | -14‧27 (-23‧83 to -4‧71) | 0‧003 |
| K72, Hepatic failure, not elsewhere classified | 358‧5 (323‧1–395‧6) | -0‧22 (-3‧53 to 3‧09) | 0‧90 | -0‧74 (-4‧40 to 2‧92) | 0‧69 |
| K74, Fibrosis and cirrhosis of liver | 40‧6 (38‧8–74‧9) | -15‧15 (-20‧03 to -10‧28) | <0‧0001 | -15‧94 (-21‧67 to -10‧20) | <0‧0001 |
| K75, Other inflammatory liver diseases | 26‧3 (22‧8–34‧5) | -5‧37 (-11‧86 to 1‧13) | 0‧11 | -6‧99 (-13‧59 to -0‧38) | 0‧04 |
| K76, Other diseases of liver | 49‧2 (47‧7–59‧8) | -2‧20 (-6‧54 to 2‧14) | 0‧32 | -3‧73 (-8‧38 to 0‧93) | 0‧12 |
| **K80-K83, Disorders of gallbladder and biliary tract** | 20‧8 (15‧5–23‧4) | -7‧34 (-12‧18 to -2‧51) | 0‧003 | -7‧69 (-13‧00 to -2‧38) | 0‧005 |
| **Q44, Congenital malformations of gallbladder, bile ducts and liver** | 26‧6 (23‧5–37‧2) | -12‧36 (-17‧27 to -7‧46) | <0‧0001 | -14‧40 (-20‧17 to -8‧63) | <0‧0001 |
| Q44.2 Atresia of bile ducts | 26‧1 (19‧6–36‧8) | -13‧25 (-19‧29 to -7‧21) | <0‧0001 | -15‧71 (-22‧98 to -8‧45) | <0‧0001 |
| Q44.7 Other congenital malformations of liver | 92‧6 (68‧2–114‧3) | -10‧52 (-21‧21 to 0‧18) | 0‧05 | -10‧94 (-23‧11 to 1‧24) | 0‧08 |

* Sensitivity analysis excluding years 2020–2021

Values are median (IQR). AAPC=average annual percent change.

**
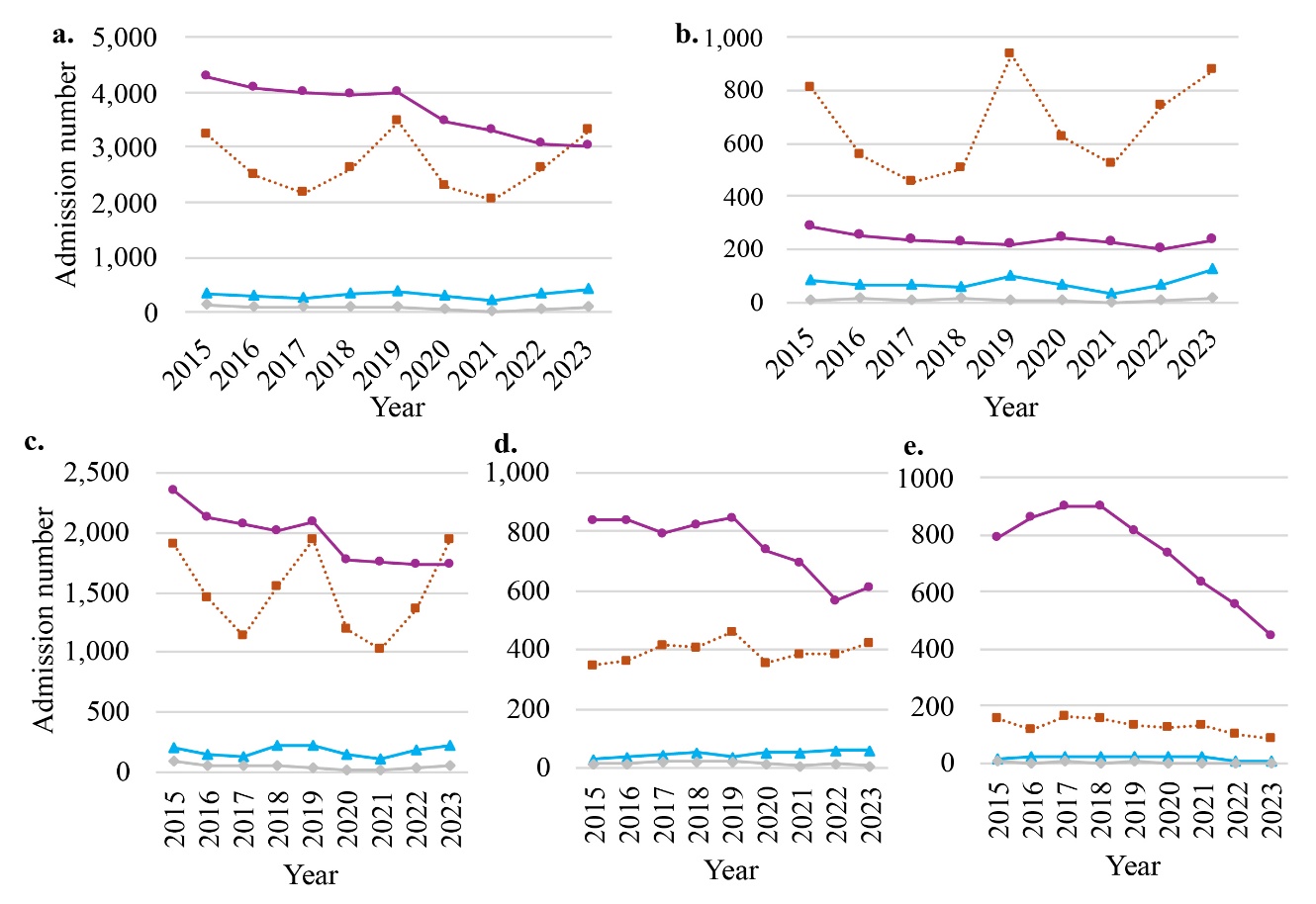
**

**Supplementary figure 1. Distribution of pediatric and adolescent hospitalizations for primary hepatobiliary diseases (a), viral hepatitis (b), diseases of liver (c), disorders of gallbladder and biliary tract (d), congenital malformation of gallbladder, bile ducts, and liver (e) stratified by hospital level**

Hospital levels are denoted by shape and color: blue triangles for primary hospitals, orange rectangles for secondary hospitals, purple circular dots for tertiary hospitals, and grey rhombus marks for private hospitals.
