# Supplementary material for: Healthcare utilisation, expenditure, and admission-based mortality associated with paediatric hepatobiliary diseases in Thailand: a national database analysis (2015–2023)
Source: Lancet Reg Health Southeast Asia. 2026 Jul 8;52:100814. doi: 10.1016/j.lansea.2026.100814 (PMC13380048; doi:10.1016/j.lansea.2026.100814)
Supplement: Equitable Partnership Declaration Form [file mmc2.docx]

**Equitable Partnership Declaration**

If any questions do not apply to your study, please indicate “N/A” for “not applicable.
For more information on how to complete this form see the Information for Authors document.

*Researcher considerations*

1. Please detail the involvement that researchers who are based in the country or countries of study had during a) study design; b) clinical study processes, such as processing blood samples, prescribing medication, or patient recruitment; c) data interpretation; and d) manuscript preparation, commenting on all aspects. If they were not involved in any of these aspects, please explain why.

*This should include a thorough description of their leadership roles in the study. Are local researchers named in the author list or the acknowledgements, or are they not mentioned at all (and, if not, why)? Please also describe the involvement of early career researchers based in the location of the study. Some of this information might be repeated from the Contributors section in the manuscript. Note: we adhere to* [*ICMJE authorship criteria*](https://www.icmje.org/recommendations/browse/roles-and-responsibilities/defining-the-role-of-authors-and-contributors.html) *for naming authors on a paper.*

| **a) Study design:** The study was conceptualized and designed by researchers based in Thailand, including the principal investigator and co-investigators affiliated with Thai academic institutions. The research question was developed to address national gaps in population-level data on pediatric hepatobiliary diseases, using locally relevant health system data. Local researchers led protocol development, variable selection, and analytical framework design. |
| --- |
| **b) Clinical study processes:** This study used a nationwide administrative database from the National Health Security Office (NHSO), Thailand. Local researchers were responsible for data acquisition, data management, and interpretation of ICD-10-TM coding structures. Although no direct patient recruitment or biological sample processing was involved, Thai investigators ensured accurate contextual understanding of healthcare delivery systems, reimbursement structures, and diagnostic coding practices. |
| **c) Data interpretation:** All data analyses were conducted and interpreted by researchers based in Thailand, including collaboration with a statistician. Interpretation of findings incorporated in-depth knowledge of the Thai healthcare system, Universal Coverage Scheme policies, and regional variations in healthcare access. Local clinicians provided disease-specific insights, particularly in pediatric hepatology. |
| **d) Manuscript preparation:** The manuscript was drafted, revised, and finalized by Thai researchers. The principal investigator supervised all stages of manuscript development. Co-authors contributed to critical revision, interpretation of findings, and contextualization within regional and global literature. All authors meet ICMJE authorship criteria and are listed accordingly. Early-career researchers were actively involved in data analysis and manuscript drafting under senior supervision. |

1. How was funding used to remunerate and enhance the skills of researchers in the countries of study? And how was funding used to improve research infrastructure at the study sites?

*Potentially effective investments into long-term skills and opportunities within local institutions could include training or mentorship in analytical techniques and manuscript writing, opportunities to lead all or specific aspects of the study, financial remuneration rather than requiring volunteers, and other professional development and educational opportunities.*

*Improvements to research infrastructure could include funding extended trial designs (eg, platform trials), establishment of long-term contracts for research staff, building research facilities, and setting up local control of funding allocation.*

| **Skills:** This study did not receive external funding. However, the research process contributed to capacity building through hands-on training in large-scale administrative data analysis, epidemiological methods, and scientific writing. Early-career researchers were mentored in statistical analysis, interpretation of population-level data, and manuscript preparation. |
| --- |
| **Research infrastructure:** Although no direct funding was allocated, the study utilized existing national data infrastructure maintained by the NHSO. Institutional support from participating universities facilitated data access, secure data handling, and analytical workflows, contributing to sustained research capacity within Thailand. |

1. How did you safeguard the researchers who implemented the study?

*Please describe how you guaranteed safe working conditions for study staff, including provision of appropriate personal protective equipment, protection from violence, and prevention of overworking.*

| All research activities were conducted in secure institutional environments. As the study involved secondary data analysis without direct patient contact, there were no risks related to biological exposure or fieldwork. Data were handled in accordance with institutional and national data protection regulations. Workload distribution was managed within the research team to avoid overburdening individual members, and all contributors participated voluntarily within their professional roles. |
| --- |

*Benefits to the communities and regions of study*

1. How does the study address the research and policy priorities of its location?

*How were the local priorities determined and then used to inform the research question? Who decided which priorities to take forward? Which elements of the study address those priorities?*

| This study addresses a critical gap in national epidemiological data on pediatric hepatobiliary diseases in Thailand. Research priorities were determined by Thai investigators based on clinical experience, national health system needs, and the absence of population-level evidence in the region. The findings provide insights into healthcare utilization, expenditure, and mortality, which are directly relevant to policy planning, resource allocation, and strengthening pediatric liver care services within Thailand. |
| --- |

1. How will research products be shared in the community of study?

*For instance, will you be providing written or oral layperson summaries for non-academic information sharing? Will study data be made available to institutions in the region(s) of study?* The Lancet Group *encourages authors to translate the summary (abstract) into relevant languages after paper editing; do you intend to translate your summary?*

| Findings from this study will be disseminated through peer-reviewed publication and academic presentations within Thailand. The authors intend to share results with clinicians, policymakers, and academic institutions. Lay summaries may be developed for broader dissemination. Data access is governed by the NHSO; however, aggregated findings will be made available to relevant stakeholders. The authors are willing to support translation of the abstract into Thai to enhance accessibility for local audiences. |
| --- |

1. How were individuals, communities, and environments protected from harm?
   1. *How did you ensure that sensitive patient data were handled safely and respectfully? Was there any potential for stigma or discrimination against participants arising from any of the procedures or outcomes of the study?*

| All data were de-identified prior to analysis. No personally identifiable information was accessible to the investigators. Data handling complied with the Thai Personal Data Protection Act (PDPA) and NHSO regulations. The study posed no risk of stigma or discrimination to individuals, as analyses were conducted at aggregate levels. |
| --- |

- 1. *Might any of the tests be experienced as invasive or culturally insensitive?*

| No direct patient interaction or invasive procedures were involved. |
| --- |

- 1. *How did you determine that work was sensitive to traditions, restrictions, and considerations of all cultural and religious groups in the study population?*

| The study design and interpretation were led by local researchers familiar with cultural, social, and healthcare contexts in Thailand. |
| --- |

- 1. *Were biowaste and radioactive waste disposed of in accordance with local laws?*

| Not applicable, as no laboratory or clinical procedures were conducted. |
| --- |

- 1. *Were any structures built that would have impacted members of the community or the environment (such as handwashing facilities in a public space)? If so, how did you ensure that you had appropriate community buy-in?*

| No physical infrastructure or environmental modifications were involved. |
| --- |

- 1. *How might the study have impacted existing health-care resources (such as staff workloads, use of equipment that is typically employed elsewhere, or reallocation of public funds)?*

| The study used existing administrative data and did not interfere with healthcare service delivery or resource allocation. |
| --- |

1. Confirm that local ethics review was sought, and please provide the approval number. If not sought, please explain why.

| Yes. Ethical approval was obtained from:   - Ethics Committee for Human Research, Khon Kaen University (Reference: HE681602) - Human Research Ethics Committee, Faculty of Medicine Ramathibodi Hospital, Mahidol University (Reference: COA. MURA 2025/261)   Informed consent was waived due to the retrospective nature of anonymized administrative data. |
| --- |

Secondary analyses

1. Have the data analysed in your study been extracted from another source, such as a national survey, rather than being directly collected by the authors of this paper?

| **Yes.** The data analysed in this study were obtained from the National Health Security Office (NHSO) administrative database. |
| --- |

If the authors of this paper were not involved in data collection, how were the findings interpreted with sufficient contextual knowledge?

The Lancet Group *believe contextual understanding is crucial for informed data analysis and interpretation.*

| Although the authors were not involved in primary data collection, several authors are clinicians and researchers based in Thailand with direct experience in the healthcare system. This ensured appropriate contextual interpretation of findings, including understanding of coding practices, healthcare delivery structures, and policy frameworks. |
| --- |

1. Please provide the title (eg, Dr/Prof, Mr/Mrs/Ms/Mx), name, and email address of an author who can be contacted about this statement.

| Name: Busara Charoenwat Email: busarcha@kku.ac.th |
| --- |

1. Finally, please provide the title and name of an author from one country of study who has seen and approved this form.

| **Name:** Songpon Getsuwan |
| --- |


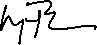


­­­­­­­­­­­­­­­­­­­­­­­­­­­­­­­­­­­­­­­­­­­______________________________________

Songpon Getsuwan
